# The molecular characterization of porcine egg precursor cells

**DOI:** 10.18632/oncotarget.18833

**Published:** 2017-06-28

**Authors:** Te-Sha Tsai, Jacqueline Johnson, Yvonne White, Justin C. John

**Affiliations:** ^1^ Centre for Genetic Diseases, Hudson Institute of Medical Research, Clayton, Victoria 3168, Australia; ^2^ Centre for Genetic Diseases, Department of Molecular and Translational Science, Monash University, Clayton, Victoria 3168, Australia; ^3^ OvaScience, Inc., Waltham, MA 02451, USA

**Keywords:** egg precursor cells, oogonial stem cells, mitochondrial DNA, mitochondrial supplementation, ageing oocyte

## Abstract

Female-factor infertility can be caused by poor oocyte quality and depleted ovarian reserves. Egg precursor cells (EPCs), isolated from the ovarian cortex, have the potential to be used to overcome female infertility. We aimed to define the origins of EPCs by analyzing their gene expression profiles and mtDNA content using a mini-pig model. We characterized FAC-sorted DDX4^+^-derived porcine EPCs by performing RNA-sequencing and determined that they utilize pathways important for cell cycle and proliferation, which supports the existence of adult mitotically active oogonial cells. Expression of the pluripotent markers *Sox2* and *Oct4*, and the primitive germ cell markers *Blimp1* and *Stella* were not detected. However, *Nanog* and *Ddx4* were expressed, as were the primitive germ cell markers *Fragilis*, *c-Kit* and *Tert*. Moreover, porcine EPCs expressed self-renewal and proliferation markers including *Myc*, *Esrrb*, *Id2*, *Klf4*, *Klf5*, *Stat3*, *Fgfr1*, *Fgfr2* and *Il6st*. The presence of *Zp1*, *Zp2*, *Zp3* and *Nobox* were not detected, indicating that porcine EPCs are not indicative of mature primordial oocytes. We performed mitochondrial DNA Next Generation Sequencing and determined that one mtDNA variant harbored by EPCs was present in oocytes, preimplantation embryos and somatic tissues over three generations in our mini-pig model indicating the potential germline origin of EPCs.

## INTRODUCTION

An increasing number of women are delaying childbirth, and, since oocyte quality declines dramatically after 35 years of age [1, 2], more women are requiring assisted reproductive treatments. Male-factor infertility can be overcome by a technique called intracytoplasmic sperm injection (ICSI), which directly injects a sperm into an oocyte, and is one of the most widely used treatments in the fertility clinic [3]. On the other hand, treatment for female-factor infertility is often restricted to *in vitro* fertilization, since ICSI does not appear to be an effective treatment for poor quality oocytes [4].

The quality of mitochondria and the numbers of copies of its genome, mitochondrial DNA (mtDNA), in oocytes are beginning to be considered, along with other factors, as indicators of oocyte quality, especially in the context of aging [5–11]. The mitochondrial genome is a highly conserved genome, which, at ∼16.6kb in size, encodes 37 of the genes that are important for functional electron transport chains that generate the vast majority of cellular ATP through oxidative phosphorylation [12, 13]. Whilst naïve, undifferentiated cells, such as pluripotent stem cells, possess a few hundred copies of mtDNA, terminally differentiated cells with high energy demands, such as neurons and cardiac muscles, possess several thousand copies [14, 15].

Low levels of mtDNA have been observed in cohorts of oocytes from couples with female-factor infertility where the oocytes fail to fertilize or arrest during pre-implantation development [7, 8, 16]. Moreover, there is evidence to suggest that this is an age-linked phenomenon as mtDNA copy number declines in oocytes with the advancement of age [17, 18]. In a pig model, we have observed mtDNA-deficiency where fertilizable oocytes have >150,000 copies of mtDNA [19, 20]. Conversely, metaphase II oocytes that are mtDNA-deficient have <100,000 copies of mtDNA, and are less likely to fertilize, or when they do they are more likely to arrest during preimplantation embryo development [19–21]. However, we have recently shown that mtDNA-deficient oocytes can be rescued by supplementation with genetically identical mitochondria, an approach known as mICSI (mitochondrial supplementation as ICSI is preformed) [22]. To this extent, blastocyst quality was significantly improved and global gene expression profiles of the resultant blastocysts closely matched those of mtDNA-normal blastocysts [22], demonstrating the beneficial effects of mitochondrial supplementation to mtDNA-deficient oocytes. Furthermore, mtDNA deficiency is not just restricted to oocytes. It has been reported in premature ovarian failure [23], ovarian insufficiency [8] and diminished ovarian reserve [24].

The number of oocytes that a female possesses, commonly known as her ovarian reserve, is generally considered to be determined at birth [25]. However, recent reports have shown the existence of mitotically active ovarian stem cells in the post-natal ovaries of mice, humans and pigs [26–28]. They are frequently referred to as egg precursor cells (EPCs) and oogonial stem cells, and have been proposed to be a source of cells to repopulate the ovary in the cases of ovarian failure. Furthermore, these cells have been used in a similar approach to mICSI, as a source of mitochondria, that has recently led to the birth of babies [29]. However, the isolation protocol for EPCs remains controversial [30–33]. Although these cells have been shown to generate fertilizable oocytes [27], and have been used to produce live offspring [34, 35], it is highly important to reproduce this protocol and characterize the resultant cells in different mammalian species in order to determine their suitability for use in assisted reproductive technologies.

The exact origins of EPCs still remains to be determined. Germ cell development is initiated from a small population of precursor cells known as primordial germ cells (PGCs), that initially express *Fragilis* (*Ifitm3*) followed by the expression of *Blimp1* (or *Prdm1*) and *Stella* (or *Dppa3*) [36, 37], which proliferate and migrate to the genital ridge during early embryo development [38]. Specified and migratory PGCs express *Ddx4* (or *Vasa*) and *Dazl* [38–40], as well as the core pluripotency genes *Oct4*, *Sox2* and *Nanog* [37, 38].

At the beginning of oogenesis, PGC possess ∼200 copies of mtDNA, which then increase to ∼2000 copies, and these are clonally replicated to reach maximal copy number in the maturing oocyte [41–43]. Consequently, any mtDNA sequence variants could be amplified to varying levels in the mature oocyte and persist into adult tissues, which we have observed in our mini-pig model [21], as this is the source of all mtDNA that is inherited in a strictly maternal fashion [44]. Whilst, pathogenic mtDNA sequence variants may lead to poor oocyte quality, many non-pathogenic variants, along with wild-type mtDNA, are likely to be transmitted across generations [21].

In the present work, we have used our established mini-pig model [21] to characterize EPCs to determine the suitability of using these cells for mitochondrial supplementation to improve oocyte quality and for transplantation into the ovary to enhance the ovarian reserve of women with low ovarian reserve, or those having undergone chemotherapy, and require ovarian transplantation. We have used the mini-pig as a model, as its embryology, development, organ systems and physiological and pathophysiological responses are more similar to those of the human than the more commonly used murine models for biomedical and pre-clinical studies [45, 46]. We used an RNA-sequencing approach to characterize EPCs, and performed in-depth analysis of mtDNA sequence variants using next-generation sequencing to determine the origins of these cells. Our work provides further insight into mammalian ovarian biology, which is important for the understanding of female fertility and ovarian ageing.

## RESULTS

### Comparison of the gene expression profiles amongst porcine, human and mouse mitotically active germ cells

In order to determine whether porcine EPCs expressed germ cell markers, we isolated putative porcine EPCs from ovarian cortex tissue and sorted the cells using an antibody specific to the DDX4 protein. In all, five cohorts of EPCs derived from the same maternal lineage were cultured for one week without passage and then underwent RNA-sequencing. We then compared their gene expression profiles with porcine PGCs, and human and mouse mitotically active germ cells that we had identified from the literature. Here, we determined that Interferon induced transmembrane protein 3 (*Ifitm3*, also known as *Fragilis*) is expressed across EPCs, porcine PGCs [47], human mitotically active germ cells [27, 48], putative porcine ovarian stem cells [28], and mouse germ line stem cells [49, 50] (Table 1). We also report that EPCs, porcine PGCs and human mitotically active germ cells expressed Telomerase reverse transcriptase (*Tert*) [27, 47, 48], which is important for stem cell self-renewal. PR/SET domain 1 (*Prdm1*, also known as *Blimp1*) and Developmental pluripotency-associated 3 (*Dppa3*, also known as *Stella*) were not expressed by EPCs, although, *Dppa3* was also not expressed by porcine PGCs [47] (Table 1).

**Table 1:** Comparison of marker gene expression between EPCs, porcine primordial germ cells, human mitotically active germ cells, porcine ovarian stem cells, and mouse germ line stem cells

Gene function	Gene code	Gene name	Porcine EPC	Porcine embryonic germ cell/primordial germ cell (Petkov 2011) [47]	Porcine ovarian putative stem cells (Bui 2014) [28]	Human mitotically active germ cells) (White 2012, Woods 2013) [27, 48]	Mouse female germ line stem cell (Xie 2014) [50]	Cultured mouse mitotically active germ cells (Imudia 2013) [49]
Primitive germ cell marker	Prdm1/Blimp1	PR/SET domain 1	no	yes	yes	yes	yes	yes
	Dppa3/Stella	Developmental pluripotency-associated 3	no	no	not determined	yes	yes	yes
	Ifitm3/Fragilis	Interferon induced transmembrane protein 3	yes	yes	yes	yes	yes	yes
	Tert	Telomerase reverse transcriptase	yes	yes	not determined	yes	not determined	not determined
Commonly used germ line marker	Dazl	DAZ Homolog	no	not determined	yes	yes	yes	yes
	Ddx4/Vasa	DEAD (Asp-Glu-Ala-Asp) box polypeptide 4	yes*	not determined	yes	yes	not determined	yes
	Kit/c-kit	KIT proto-oncogene receptor tyrosine kinase	yes	yes	yes	not determined	no	not determined
	Adad1/Tenr	Adenosine deaminase domain containing 1	yes	yes	not determined	not determined	not determined	not determined
	Sycp2	Synaptonemal complex protein 2	yes	yes	not determined	not determined	not determined	not determined
Meiosis marker	Stra8	Stimulated By Retinoic Acid 8	no	not determined	not determined	not determined	not determined	yes
	Meioc	Meiosis Specific With Coiled-Coil Domain	no	not determined	not determined	not determined	not determined	not determined
Oocyte/follicle marker	Figα	Folliculogenesis Specific BHLH Transcription Factor	no	not determined	not determined	not determined	yes	not determined
	Zp1	Zona Pellucida glycoprotein 1	no	not determined	no	yes	yes	not determined
	Zp2	Zona Pellucida glycoprotein 2	no	not determined	not determined	yes	No	not determined
	Zp3	Zona pellucida glycoprotein 3	no	not determined	not determined	yes	yes	not determined
	Nobox	NOBOX oogenesis homeobox	no	not determined	not determined	yes	no	not determined
	Gdf9	Growth differentiation factor 9	yes	not determined	no	yes	yes	not determined
Core-pluripotency marker	Sox2	SRY-Box 2	no	no	yes	not determined	no	yes
	Oct4	POU Class 5 Homeobox 1	no	no	yes	not determined	yes	yes
	Nanog	Homeobox Transcription Factor Nanog	yes*	no	yes	not determined	no	yes
Cell proliferation/sef-renewal marker	Rex1/Zfp42	ZFP42 Zinc Finger Protein	no	no	yes	not determined	no	not determined
	Myc	Proto-Oncogene C-Myc	yes	yes	yes	not determined	no	not determined
	Esrrb	Estrogen Related Receptor Beta	yes	yes	no	not determined	no	not determined
	Zfx	X-Linked Zinc Finger Protein	yes	not determined	not determined	not determined	yes	not determined
	Id2	Inhibitor Of Differentiation 2	yes	yes	not determined	not determined	no	not determined
	Klf4	Kruppel-Like Factor 4	yes	yes	yes	not determined	no	not determined
	Klf5	Kruppel-Like Factor 5	yes	yes	not determined	not determined	no	not determined
	Tbx3	T-Box Protein 3	yes	no	not determined	not determined	not determined	not determined
	Stat3	Signal Transducer And Activator Of Transcription 3	yes	yes	not determined	not determined	no	not determined
	Fgfr1	Fibroblast Growth Factor Receptor 1	yes	yes	not determined	not determined	no	not determined
	Fgfr2	Fibroblast Growth Factor Receptor 2	yes	yes	not determined	not determined	no	not determined
	Lifr/Il6st	Leukemia Inhibitory Factor Receptor	yes	yes	not determined	not determined	no	not determined
	Pparg	Peroxisome Proliferator Activated Receptor Gamma	yes	not determined	not determined	not determined	no	not determined
Cell cycle marker	Cdk1	Cyclin-dependent kinase 1	yes	not determined	not determined	not determined	yes	not determined
	Cdk2	Cyclin-dependent kinase 2	yes	not determined	not determined	not determined	yes	not determined
	Rpa1	Replication protein A1	yes	not determined	not determined	not determined	yes	not determined
	Rabgap1	RAB GTPase activating protein 1	yes	not determined	not determined	not determined	yes	not determined
	App	Amyloid beta precursor protein	yes	not determined	not determined	not determined	yes	not determined

Footnote: * indicates that gene expression was not detected by RNA-sequencing after data normalization, but was detected in RT-PCR.

We found that *Ddx4* (*Vasa*; DEAD Asp-Glu-Ala-Asp box polypeptide 4) was expressed by EPCs, as determined by reverse transcription PCR (RT-PCR) and Sanger sequencing (Supplementary Data 1A and 1B, respectively). Other commonly used germ line markers including KIT proto-oncogene receptor tyrosine kinase (*Kit* or *c-kit*), Adenosine deaminase domain containing 1 (*Adad1* or *Tenr*) and Synaptonemal complex protein 2 (*Sycp2*), were expressed by both EPCs and porcine PGCs [47], which further demonstrates the similarity between these two populations (Table 1). To assess whether meiosis is initiated in EPCs, we found that Stimulated By Retinoic 8 (*Stra8*) is not expressed by EPCs, but is expressed in mouse mitotically active germ cells [49] (Table 1). Likewise, the meiotic marker, Meiosis Specific With Coiled-Coil Domain (*Meioc*) is not expressed by EPCs (Table 1). Both populations did not express oocyte markers NOBOX oogenesis homeobox (*Nobox*), Zona pellucida glycoproteins 1 to 3 (*Zp1* to *3*), or Folliculogenesis Specific BHLH Transcription Factor (*Figα*), and only EPCs expressed Growth differentiation factor 9 (*Gdf9*) [47] (Table 1). Both EPCs and porcine PGCs did not express pluripotency markers SRY-Box 2 (*Sox2*) or POU Class 5 Homeobox 1 (*Oct4*), which differs to putative pig ovarian stem cells and cultured mouse mitotically active germ cells (Table 1) [28, 49]. However, when we performed RT-PCR, we detected expression of the Homeobox Transcription Factor Nanog (*Nanog*) in EPCs (Table 1 and Supplementary Data 1A).

To determine the cell proliferation and self-renewal potential of EPCs, we assessed markers such as Proto-Oncogene C-Myc (*Myc*), Estrogen Related Receptor Beta (*Esrrb*), Inhibitor Of Differentiation 2 (*Id2*), Kruppel-Like Factor 4 (*Klf4*), Kruppel-Like Factor 5 (*Klf5*), Signal Transducer And Activator Of Transcription 3 (*Stat3*), Fibroblast Growth Factor Receptor 1 (*Fgfr1*), Fibroblast Growth Factor Receptor 2 (*Fgfr2*), and Leukemia Inhibitory Factor Receptor (*Lifr*), and found that they were commonly expressed by EPCs and porcine PGCs [47] (Table 1). Moreover, EPCs expressed cell cycle markers Cyclin-dependent kinase 1 (*Cdk1*), Cyclin-dependent kinase 2 (*Cdk2*), Replication protein A1 (*Rpa1*), RAB GTPase activating protein 1 (*Rabgap1*) and Amyloid beta precursor protein (*App*) (Table 1), which suggests that EPCs are mitotically active.

### Gene ontology using the PANTHER classification system

The entire list of normalized RNA-sequencing data, which consisted of 13806 genes, was then analyzed using the “Gene List Analysis” tool, from the Gene Ontology Consortium database. Here, we report that the top five biological functions for EPCs were cellular process (GO:0009987; 3891/13175 genes), metabolic process (GO:0008152; 3640/13175 genes), localization (GO:0051179; 1066/13175 genes), cellular component organization or biogenesis (GO:0071840; 923/13175 genes), and response to stimulus (GO:0050896; 865/13175) (Supplementary Data 2A and 2B, respectively).

**Figure 1:**
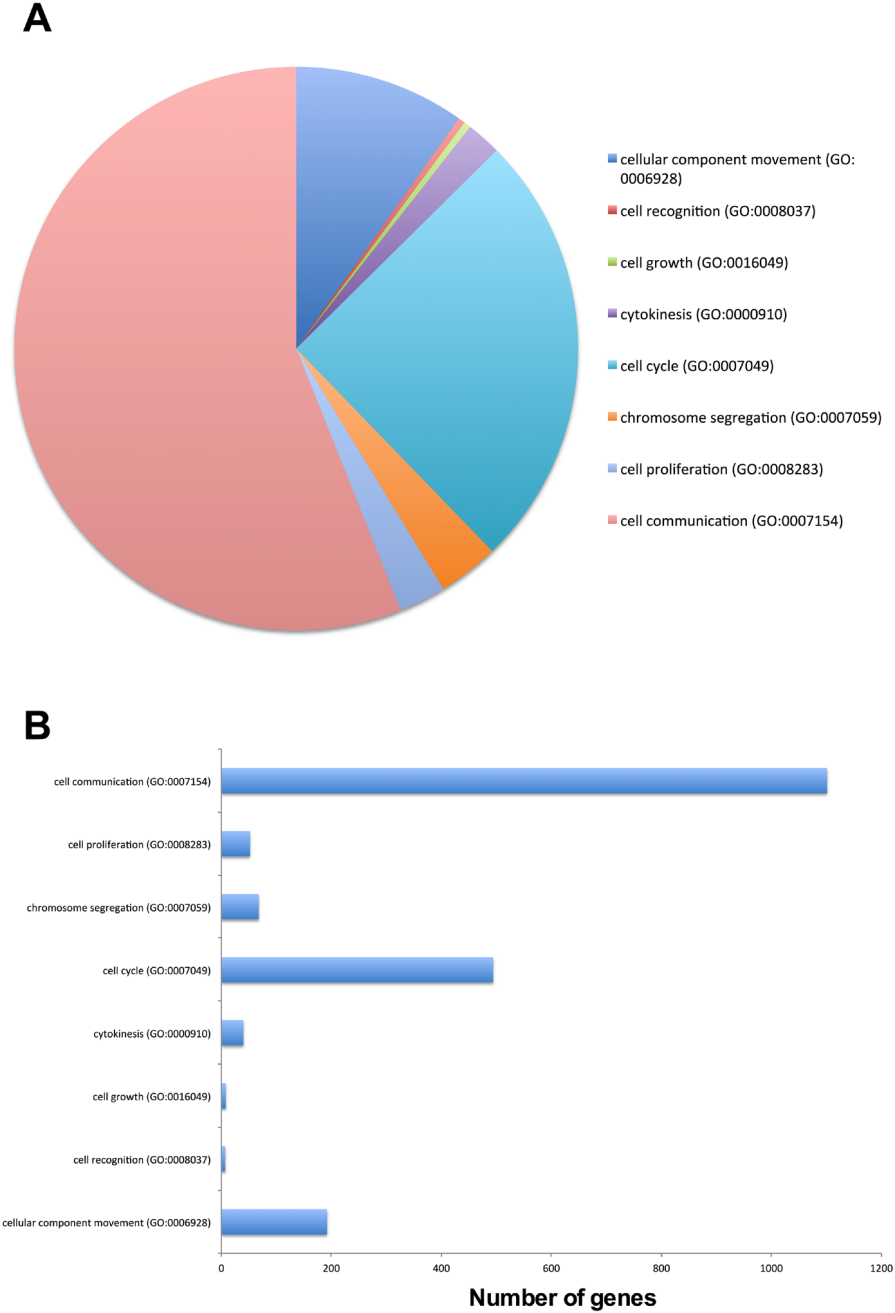
Top cellular functions of EPCs, as determined by the PANTHER classification system from the Gene Ontology Consortium database **(A)**, and the number of genes involved **(B)**.

Within the cellular process category, the functions of those genes were further determined (Figure 1). The top five cellular functions (Figure 1A) and the number of genes involved (Figure 1B) were cell communication (GO:0007154; 1101/1962 genes), cell cycle (GO:0007049; 494/1962 genes), cellular component movement (GO:0006928; 192/1962 genes), chromosome segregation (GO:0007059; 68/1962 genes), and cell proliferation (GO:0008283; 52/1962 genes). Together, these data demonstrate that EPCs have the propensity to be mitotically active and proliferate.

### Top canonical pathways and cellular functions utilized by EPCs

To further elucidate the gene expression profiles, we used another bioinformatics analytical software tool, Ingenuity Pathway Analysis (IPA). We determined the major canonical pathways utilized by EPCs using the ‘Core analysis’ tool. The top five canonical pathways were EIF2 signaling, which plays a role in protein synthesis (P=7.30×10^−45^); regulation of eIF4 and p70S6K signaling, which is critical for translational regulation (P=1.43×10^−34^); mTOR signaling, which is involved in cell survival and proliferation (P=9.30×10^−29^); the protein ubiquitination pathway, which plays a role in the degradation of short lived regulatory proteins (P=6.79×10^−27^); and the PI3K/AKT signaling pathway, which plays a central role in signal transduction pathways of cytokines, growth factors and other extracellular matrix proteins (P=4.80×10^−20^) (Supplementary Data 3 and 4). These pathways are important since protein synthesis is essential for germline stem cells to continue proliferation, to differentiate or to enter apoptosis [51].

The IPA ‘Core Analysis’ tool also determined that the top molecular and cellular functions of EPCs are: cell death and survival (from 1651 genes; P=1.59×10^−09^ to 4.78×10^−96^), cellular growth and proliferation (from 1814 genes; P=5.50×10^−10^ to 3.42×10^−84^), gene expression (from 1136 genes; P=1.38×10^−11^ to 3.75×10^−70^), protein synthesis (from 526 genes; 2.03×10^−13^ to 1.68×10^−64^) and RNA post-transcriptional modification (from 222 genes; P=4.83×10^−16^ to 1.49×10^−62^). Moreover, the top predicted developmental functions are: organismal survival (from 1130 genes; P=1.49×10^−14^ to 1.42×10^−74^), embryonic development (from 837 genes; P=8.04×10^−10^ to 1.60×10^−34^), organismal development (from 1293 genes; P=1.29×10^−9^ to 1.60×10^−34^), tissue morphology (from 611 genes; P=6.84×10^−10^ to 1.60×10^−34^) and cardiovascular system development and function (from 650 genes; 1.73×10^−9^ to 2.09×10^−28^) (Supplementary Data 4). These predicted functions indicate that EPCs have the transcripts to support early embryo development.

### Top gene regulation networks utilized by EPCs

The top four gene networks of that were identified by IPA to be significantly utilized (network score ≥30) were those involved in: connective tissue development, cancer, cell death and survival, and gene expression (Table 2). An important caveat for interpreting IPA network analysis is that its results mainly focus on diseases and functions. However, pathways involved in cell death and survival and cancer are also often utilized by, for example, stem cells [52]. Overall, the top eleven networks (network score ≥28), showed that EPCs utilized pathways that are important for cell morphology, cellular assembly and organization, cell to cell signaling, cell growth and proliferation, and cellular development (Table 2). These results are consistent with the top biological functions determined by PANTHER and the major canonical pathways determined by IPA.

**Table 2:** Top gene networks utilized by EPCs as determined by Ingenuity Pathway Analysis

Top diseases and functions	Molecules in network	IPA score	Focus molecules
Connective Tissue Development and Function, Developmental Disorder, Hereditary Disorder	ADRBK1, ARGLU1, C1D, CDKN2AIPNL, DCAF10, ENOPH1, ESCO1, ETFA, ETFDH, EXOSC7, EXOSC9, FAM133B, FOCAD, HEXA, HEXB, HPS5, KIAA2013, MAK16, MRPS35, NCS1, NNT, PAPD7, PAPSS1, PAPSS2, RMND5A, SLAIN2, SMC5, SMC6, SMYD5, SS18L2, TMEM132A, TSNAX, TUFT1, WAC, YPEL5	30	35
Cancer, Hematological Disease, Immunological Disease	ABL1, ARL5A, ATIC, BOD1L1, CBX3, CHD4, CNBP, DDX47, DEGS1, DHX15, DPY19L1, EIF5B, FJX1, FUBP1, GART, HDGF, KDM3B, KDM5B, KPNA2, LMNB2, MTF2, NCBP1, PAICS, PLS3, PRPS1, PSIP1, RBBP4, RCOR1, RECQL, SETX, SLC16A1, STK38, SUB1, ZDHHC16, ZNF217	30	35
Cell Death and Survival, Infectious Diseases, Gene Expression	AMBRA1, ANP32B, ATF7IP, BUB1, CPEB2, EEF2, ERCC3, GANAB, HSDL2, HSP90AA1, HUWE1, KCTD2, KDM4B, KIF1B, LRRC42, MACF1, MAST2, MXRA7, NANS, NUF2, PCBP1, PCMT1, PPIG, PSMA1, PSMA3, PSMC1, PSMC3, PSMC5, PSMD2, RAD23B, RALBP1, RNASEH2C, SNTB2, TFE3, TNIK	30	35
Gene Expression, Connective Tissue Disorders, Developmental Disorder	ACER3, ANO6, CCDC25, CDR2L, CHSY1, COQ10B, DCUN1D4, EFCAB14, ELAVL1, ERMP1, FAM105A, HECA, IER3IP1, ISOC1, MEX3D, MGAT2, PCNP, PDZD8, PEX19, PITHD1, RAP2C, S100PBP, SELT, SLC10A3, SLC18B1, SMIM7, SPPL3, TM7SF3, TMCO1, TMEM123, TMEM167B, TMEM41B, TMX1, ZNF521, ZNF664	30	35
Cell Morphology, Cellular Assembly and Organization, Cellular Function and Maintenance	60S ribosomal subunit, ABCF1, BMS1, CBY1, CEP164, DDX24, DNTTIP2, DZIP1, FRYL, GALK1, GNL2, GRK5, KIAA0930, MRTO4, MYBBP1A, NIFK, NLE1, NMD3, NSA2, OTUD4, PAK1IP1, PEF1, PTCD3, PUM3, RPF1, RPL8, RPL14, RPL26L1, RPL7L1, RRP8, RSL24D1, STAU2, USP36, UTP18, WDR3	28	34
Cancer, Cell Death and Survival, Organismal Injury and Abnormalities	EBNA1BP2, GNRH, GRSF1, HAUS2, KRR1, MRPL3, MRPL24, MRPL38, MRPL40, MRPL46, MRPS6, MRPS7, MRPS9, MRPS10, MRPS22, MRPS26, MRPS27, MRPS34, NEMF, PREP, RANBP6, RPL6, RPL13, RPL15, RPL17, RPL26, RPL27, RPL34, RPL38, RPL27A, RPS8, SMIM20, SRSF9, SUCO, TRA2A	28	34
Cellular Assembly and Organization, Cell-To-Cell Signaling and Interaction, Reproductive System Development and Function	ACTR1B, AHI1, CCT2, CCT3, CCT4, CCT5, CCT7, CCT8, CCT6A, CIPC, DCAF7, DENND4C, DOCK5, DSP, ECD, HSF2, MAPK9, NMT1, PDCD10, Ppp2c, PPP2CB, PPP2R1A, PPP2R1B, PPP2R2A, PPP2R5C, RABGEF1, SIRT2, STK24, STK25, STRN, SUN2, TCP1, TRMT112, TXNDC9, UNC45A	28	34
Cardiovascular Disease, Connective Tissue Disorders, Developmental Disorder	ANKIB1, ATG2B, ATP8B2, CCDC50, CDIP1, CTTNBP2NL, DCHS1, DENR, DHRS7, FAR1, FARSA, GRAMD1A, HECTD1, ITM2B, Lamin, LRRC57, MFAP3, MRPL49, NUP155, NUTF2, OTUD7B, R3HDM4, RNF19B, RYK, SBF2, STRN4, TALDO1, TMEM59, TMEM30A, TMEM59L, TXNL1, UBC, ZDHHC20, ZFAND3, ZRANB1	28	34
Cell Signaling, Gene Expression, Cellular Function and Maintenance	ACADVL, CAD, CBL, CDK9, CHD1, CNN1, DCTN3, DECR1, FLOT1, FOXP4, HMMR, KLHL12, LRPPRC, MED4, MED8, MED12, MED16, MED17, MED25, MED28, mediator, MMS19, NIPBL, OSTF1, POLR2A, QKI, RPLP2, RUVBL2, SART3, SKIV2L2, THRAP3, TRRAP, TXLNA, TXLNG, ZW10	28	34
Small Molecule Biochemistry, Post-Translational Modification, Lipid Metabolism	APPBP2, BNIP3, CACFD1, Ces, COMT, CREB3, CYP51A1, DAD1, EBP, ENC1, FAM213A, FAM3A, FIS1, HSD3B1, IFRD1, IMPDH1, MFSD7, MFSD11, MSMO1, NFE2L2, NUCB2, OAF, OAT, ORMDL1, SLC39A13, SLC41A2, SPTSSA, ST3GAL4, TBC1D15, TMEM115, TMEM230, TPI1, UGGT2, UNC50, VKORC1	28	34
Cell Death and Survival, Cellular Development, Cellular Growth and Proliferation	ABRACL, ANXA2, BTG3, CCPG1, CDC37, CEP290, CTNND1, CUL2, DCTN1, EWSR1, Fgf, FGF11, FUS, GLS, HLTF, MAOB, MET, NAP1L3, NRP1, PKM, PRPF19, RARA, RBPJ, RCC1, SDC1, SMARCA4, SNW1, SSB, SUZ12, TFIP11, TNC, UPF1, VCP, WRNIP1, YBX1	28	34

### Top upstream regulators that determine EPC gene expression

Upstream regulators are master molecules that target and regulate gene expression in EPCs. We have identified these upstream molecules to provide further support to our biological function analysis. Here, we identified 477 upstream regulators that activate, and 246 that inhibit EPC gene expression (Supplementary Data 5). The activating upstream regulators, as determined by IPA, included 87 transcription regulators, 29 growth factors, 7 nuclear receptors and 24 cytokines. All regulators identified have a z-score of >2 or <2 and were ranked from the lowest to highest P-value (all <0.05) (Supplementary Data 5).

The top ten activating transcription regulators were v-myc avian myelocytomatosis viral oncogene homolog (*Myc*), tumor protein p53 (*Tp53*), hepatocyte nuclear factor 4 alpha (*Hnf4a*), v-myc avian myelocytomatosis viral oncogene neuroblastoma derived homolog (*Mycn*), X-box binding protein 1 (*Xbp1*), nuclear factor, erythroid 2 like 2 (*Nfe2l2*), hypoxia inducible factor 1 alpha subunit (*Hif1a*), huntingtin (*Htt*), E2F transcription factor 1 (*E2f1*), and Fos proto-oncogene, AP-1 transcription factor subunit (*Fos*) (Table 3). These genes are involved in the biological functions of cell proliferation, cell cycle regulation, cellular response to stress and nutrient, and maintenance of cell homeostasis (Table 3).

**Table 3:** Upstream regulators that positively regulate EPC gene expression as determined by Ingenuity Pathway Analysis

Molecule type	Upstream regulator	Biological function	No. of target genes	P-value	Z-score
Transcription Regulator	MYC	Cell proliferation, cell cycle regulation	457	2.32E-83	8.884
	TP53	Cell cycle regulation	563	2.23E-82	4.559
	HNF4A	Glucose homeostasis, lipid homeostasis	728	1.51E-81	2.167
	MYCN	Cell proliferation	165	2.67E-60	2.925
	XBP1	Cellular response to nutrient, cell growth	124	5.36E-40	9.837
	NFE2L2	Cellular response to stress	166	1.56E-26	11.009
	HIF1A	Cellular response to hypoxia	147	3.55E-20	8.085
	HTT	Regulation of mitochondrial function	232	6.71E-20	4.924
	E2F1	Cell cycle regulation	168	9.48E-20	4.241
	FOS	Cellular response to stimulus	187	1.12E-18	2.507
Nuclear Receptor	ESR1	Ovarian follicle growth	438	9.49E-41	5.358
	PGR	Cellular response to gonadotropin	110	1.53E-16	6.862
	PPARG	Lipid metabolism, glucose homeostasis	139	9.64E-10	3.756
	PPARA	Glucose metabolism, fatty acid metabolism	116	9.18E-06	4.608
	AR	Cell growth and proliferation	116	2.05E-05	6.679
	ESRRA	Cell proliferation	53	1.86E-04	5.735
	ESRRG	Cell proliferation	14	7.26E-03	3.121
Growth Factor	TGFB1	Cell growth and proliferation, migration	550	1.54E-49	10.924
	HGF	Cell proliferation migration	180	8.4E-21	8.682
	ANGPT2	Cell differentiation, germ cell development	85	2.84E-16	6.092
	VEGFA	Cell migration, angiogenesis	102	2.05E-14	7.275
	EGF	Potent mitogenic factor	159	7.61E-13	8.927
	TGFB3	Cell growth and proliferation	48	9.23E-11	5.633
	AGT	Extracellular matrix organization	136	5.25E-10	7.643
	IGF1	Cellular response to insulin and glucose	120	4.03E-08	6.814
	FGF2	Cell division and proliferation	106	2.65E-07	6.851
	KITLG	Germ cell development, ovarian follicle development	71	4.97E-06	5.675

The top seven positive nuclear-receptor regulators were estrogen receptor 1 (*Esr1*), progesterone receptor (*Pgr*), peroxisome proliferator activated receptor gamma (*Pparg*), peroxisome proliferator activated receptor alpha (*Ppara*), androgen receptor (*Ar*), estrogen related receptor alpha (*Esrra*), and estrogen related receptor gamma (*Esrrg*). These ligand-regulated transcription factors play key roles in regulating cell growth and proliferation, lipid and glucose metabolism and follicular growth (Table 3).

The top ten growth factors were transforming growth factor beta 1 (*Tgfb1*), hepatocyte growth factor (*Hgf*), angiopoietin 2 (*Angpt2*), vascular endothelial growth factor A (*Vegfa*), epidermal growth factor (*Egf*), transforming growth factor beta 3 (*Tgfb3*), angiotensinogen (*Agt*), insulin like growth factor 1 (*Igf1*), fibroblast growth factor 2 (*Fgf2*), and KIT ligand (*Kitlg*). These growth factors are important for germ cell development, cell proliferation, cell metabolism and cell migration (Table 3).

Upstream regulatory molecules that inhibit EPC gene expression included 27 transcription regulators, 1 growth factor, 42 mature microRNAs and 27 microRNAs (Supplementary Data 5). The top ten inhibiting transcription regulators were nuclear protein 1 (*Nupr1*), promyelocytic leukemia (*Pml*), cyclin dependent kinase inhibitor 2A (*Cdkn2a*), Kruppel like factor 3 (*Klf3*), SMAD family member 7 (*Smad7*), lysine demethylase 5A (*Kdm5a*), lysine demethylase 5B (*Kdm5b*), SAM pointed domain containing ETS transcription factor (*Spdef*), interferon regulatory factor 4 (*Irf4*), and MAX interactor 1 (*Mxi1*) (Table 4). These transcription regulators are regulators of cell cycle, cell proliferation, chromatin organization and germ cell migration (Table 4). We also identified microRNAs that are likely important in the regulation of self-renewal in EPCs, specifically those that regulate *Oct4*, *Klf4* and *Myc* (Table 4). Indeed, microRNAs have been reported to play regulatory roles in stem and germ cells [53].

**Table 4:** Upstream regulators that negatively regulate EPC gene expression as determined by Ingenuity Pathway Analysis

Molecule type	Upstream regulator	Biological function	No. of target genes	P-value	IPA Z-score	Reference (DOI)
Transcription Regulator	NUPR1	Cell cycle	166	3.09E-14	−3.035	IPA Knowledge database
	PML	Regulation of the TGF-beta signaling pathway	58	2.22E-12	−3.195	IPA Knowledge database
	CDKN2A	Cell cycle negative regulator	100	3.37E-11	−2.147	IPA Knowledge database
	KLF3	Multicellular organismal development	112	1.48E-10	−7.504	IPA Knowledge database
	SMAD7	Negative regulation of BMP signaling pathway, negative regulation of cell migration	53	8.29E-10	−4.682	IPA Knowledge database
	KDM5A	Chromatin modification, chromatin organization	54	2.02E-09	−5.900	IPA Knowledge database
	KDM5B	Chromatin modification	55	3.34E-09	−4.673	IPA Knowledge database
	SPDEF	Germ cell migration	33	3.00E-08	−4.402	IPA Knowledge database
	IRF4	Interferon-gamma-mediated signaling pathway	46	1.48E-04	−4.662	IPA Knowledge database
	MXI1	Negatively regulate MYC function	10	1.51E-04	−2.919	IPA Knowledge database
Mature MicroRNA	miR-124-3p	Potential regulator of PIM1	118	1.36E-28	−10.788	Deng et al. 2016 (10.1111/cas.12946)
	miR-16-5p	Potential regulator of SMAD3	102	1.23E-24	−9.938	Li et al. 2015 (10.2174/1381612821666150909094712)
	miR-1-3p	Unknown	99	5.58E-24	−9.767	n/a
	let-7a-5p	Potential regulator of CCND1 and MYC	78	7.77E-20	−8.622	Ghanbari et al. 2015 (10.4137/BIC.S25252)
	miR-30c-5p	Potential regulator of EIF2A	63	2.14E-19	−7.805	Jiang et al. 2016 (10.1038/srep21565)
	miR-155-5p	Potential regulator of DNMT1	73	1.24E-15	−8.437	Zhang et al. 2015 (10.1093/nar/gkv518)
	miR-483-3p	Potential regulator of CDC25A	25	2.50E-08	−4.969	Bertero et al. 2013 (10.1038/cdd.2013.5)
	miR-133a-3p	Potential regulator of RBPJ	27	5.27E-08	−5.065	Huang et al. 2016 (ISSN:2156-6976/ajcr0040390/; PMID: 27904763)
	miR-145-5p	Potential regulator of OCT4 and KLF4	29	8.90E-08	−5.312	Xu et al. 2009 (10.1016/j.cell.2009.02.038)
	miR-29b-3p	Potential regulator of TGFB1	29	5.48E-07	−5.260	Lu et al. 2016 (10.1096/fj.201600722R)

### mtDNA copy number and expression of *Polg*

EPCs from the current work were harvested from ovaries of mini-pigs from a established colony originating from a single founder female [21], which ensures that each of the offspring inherits the same population of mtDNA. We firstly determined that the mtDNA copy number of EPCs (1131 ± 411, mean ± SEM) was significantly lower than immature oocytes (Supplementary Data 6) and is within the range for PGC mtDNA copy number [41–43, 54]. We then determined the expression levels of mitochondrial specific polymerase gamma (*Polg*) in mini-pig heart, muscle and EPCs, and found that EPCs express significantly fewer transcripts than heart tissues (Supplementary Data 7). These data show that EPCs maintain low mtDNA copy number, which is indicative of their naïve state.

### mtDNA sequence variants harbored by the EPCs

To determine the number of positions within the mitochondrial genome that harbored a sequence variant, we performed in depth Next Generation Sequencing with >4000 times coverage. MtDNA sequence variants that were harbored between 3 to 50% were compared amongst EPCs, oocytes, 2-cell embryos, 4-cell embryos, 8-cell embryos and ovarian tissues (Table 5). Eighteen positions within the mitochondrial genome were affected, with the mean number of variants harbored by EPCs, oocytes, 2-cell embryos, 4-cell embryos, 8-cell embryos and ovarian tissues being 7.3, 2.9, 3.5, 3, 3 and 2.6, respectively (Table 5). EPCs harbored the most number of variants, whilst ovarian tissues had the least. The variant A376del was harbored by all samples and at a mean frequency of 4.6 ± 0.1% (mean ± S.E.M), as was A5188del at a mean frequency of 4.8 ± 0.08% (mean ± S.E.M). This indicates that some variants that are present at low levels can persist from oogenesis through embryo development to adulthood. Moreover, the variant T7317C was harbored only by EPCs and oocytes and this variant was harbored at relatively high frequencies. This demonstrates that EPCs and oocytes possessed a similar population of mtDNA. However, the T7317C variant is eliminated post-fertilization but persists in putative germline cells. The A1253del variant was also found across all groups, but found less frequently in EPCs (25%) compared with oocytes (65%), embryos (100%) and ovarian tissues (60%).

**Table 5:** Mitochondrial DNA sequence variants in EPCs, oocytes, 2-cell embryos, 4-cell embryos, 8-cell embryos and ovarian tissues

Position	WT	V	Gene	EPC				Immature oocytes																	2 cell		4 cell		8 cell		Ovarian tissue				
				A1	A2	A3	A4	L1	L2	L3	L4	L5	L6	L7	L8	L9	L10	L11	L12	L13	L14	L15	L16	L17	E13	E14	E18	E19	E23	E24	O1	O2	O3	O4	O5
376	A	-	12s RNA	3.2	3.3	3.4	4.2	4.5	4.5	4.0	4.8	4.7	4.7	4.8	4.6	4.8	5.0	5.1	5.0	4.7	4.9	5.8	5.5	4.7	5.2	4.7	4.9	5.5	4.8	4.8	5.1	4.6	4.5	4.4	4.0
960	T	C	12s RNA																						7.0										
1253	A	-	16s RNA	9.3				3.2					3.1		3.1	3.1	3.4	3.4	3.6	3.4	3.4	3.6		3.2	3.4	3.3	3.4	3.6	3.6	3.3	3.5	3.0	3.4		
1497	-	A	16s RNA							8.2																									
3256	G	A	NADH1				3.8																												
3495	A	G	NADH1		8.8																														
4932	C	T	NADH2		4.2																														
5188	A	-	Origin of L-strand replication	3.5	4.7	4.6	4.0	5.0	4.4	5.1	5.3	5.1	4.9	4.7	4.6	4.8	4.8	5.0	5.2	4.7	5.0	4.7	4.2	4.4	5.6	4.6	5.1	4.8	4.6	4.4	5.1	5.2	5.4	5.0	4.4
7317	T	C	COII	8.2	4.3		12.8							5.1		3.7			7.7		19.3														
12101	C	T	NADH5				4.5																												
12535	T	A	NADH5		4.2																														
12860	A	G	NADH5			3.4																													
15760	T	C	Control region			9.5																													
16022	T	C	Control region	3.7																															
16140	A	G	Control region		6.9	4.9	3.1																												
16142	A	G	Control region		6.7	4.6																													
16352	A	G	Control region	5.0	7.5	4.6																													
16561	A	G	Control region		4.0																														

Footnote: WT = wild type; V = variant.

We then compared the variants with those that we had previously identified in our mini-pig model [21]. The A376del was harbored by oocytes, preimplantation embryos and somatic tissues, and was maintained in our mini-pig colony over three generations, which was derived from one common maternal ancestor. Specifically, in the immature (∼10%) and mature (∼20%) oocytes and embryos (∼50%), the levels of A376del were very different. However, in somatic tissues the variant was present at low levels (<20%) across three generations in all tissues examined. This suggests that this variant is present in the germline and is regulated at different stages of development in the offspring [21].

## DISCUSSION

Mitochondrial supplementation, otherwise known as mICSI, is a relatively new assisted reproductive technique that has the potential to have a significant impact on the treatment of female-factor infertility. This technique arose from the concept of ooplasmic transfer from younger to older women as a means to rescue poor quality oocytes [55]. To perform mICSI, purified mitochondria without accompanying mRNA and other cellular factors are injected into the cytoplasm of metaphase II oocytes along with the spermatozoa during the process of ICSI [22]. The technique of ICSI has been performed for nearly three decades and has led to the birth of over 2.5 million children [3]. Whilst ICSI has successfully treated male-factor infertility, especially those associated with poor or abnormal semen quality, it does not improve pregnancy outcomes for women over the age of 40 [4] or for mtDNA deficiency [22].

We have previously shown that by performing mICSI in our porcine model of mtDNA-deficient oocytes, supplementation of 800 copies of mtDNA resulted in a significant (4.4 fold) increase of mtDNA copy number at the 2-cell embryo stage [22]. This is important as it ensures sufficient copies are allocated to each blastomere as they divide [15]. Moreover, the global gene expression profiles of the resultant blastocysts from mtDNA-deficient oocytes were enhanced to resemble blastocysts derived from mtDNA-normal oocytes [22]. However, the mitochondria isolated from our previous work were derived from metaphase II oocytes. The present work assesses the suitability of utilizing EPCs for mitochondrial supplementation by determining their origins through their gene expression profiles and the mtDNA variants they harbor. As a consequence, this will also define their suitability for transplantation purposes to restore ovarian function for women with, for example, premature ovarian failure.

Here, we have cultured isolated EPCs for one week, without passage, and observed that they were not dormant and were able to proliferate under *in vitro* conditions. We then assessed the gene expression profiles of EPCs, and found that they shared some key markers with porcine PGCs [47]. They express primitive germ cell specific markers *Fragilis* [36] and *Tert*, which is the enzymatic component of telomerase and is highly expressed in germline stem cells [56]. The expression of *Fragilis* and *Tert* was also found in human mitotically active post-natal germ cells [27, 48]. Interestingly, *Fragilis* was the only primitive germ cell marker that was consistently detected in mouse and pig putative germline stem cells [28, 50]. However, we did not observe the expression of *Blimp1* or *Stella*, which are other primitive germ cell markers [36, 57]. We argue that since *Blimp1* and *Stella* are only expressed in a small proportion of *Fragilis* positive cells [36, 57], the gene expression levels in the isolated porcine EPCs may be very low. Nevertheless, the expression of *Ddx4*, which encodes for the evolutionarily conserved and germ cell specific VASA protein [39, 58], was detected in the EPCs, albeit at low levels.

The discovery of mitotically active ovarian stem cells has challenged the widely accepted view that the ovarian reserve is fixed at birth (approximately 1 million follicles) and cannot be renewed [25, 59, 60]. The existence of ovarian stem cells was initially reported in mice [26], and subsequently found in human ovarian cortical tissues [27]. Since then putative ovarian stem cells have been isolated by multiple groups and in several species [28, 49, 50]. Ovarian stem cells have the capacity to proliferate, differentiate to oocyte-like cells and can be fertilized to produce live offspring [27, 34, 35]. We chose to use cells that had been cultured in order to undertake our analysis on cells that had the propensity to proliferate and were not trapped in a dormant state, which is a key characteristic that we would expect from cells with the potential to give rise to more mature cell types. However, they may change their characteristics or selected for particular sub-groups.

In the present work, of the core pluripotency markers, EPCs only expressed *Nanog* but not *Sox2* or *Oct4*. However, cell proliferation and self-renewal markers such as *Myc*, *Esrrb*, *Zfx*, *Id2*, *Klf4*, *Klf5*, *Tbx3*, *Stat3*, *Fgfr1*, *Fgfr2*, *Lifr*, and *Pparg* were expressed. It is important to note that expression of the pluripotency network genes varies between species, as demonstrated in human and mouse embryonic stem cells [61]. Therefore, extrapolation of results from pig, mouse and human should be taken with caution. Nevertheless, the cell cycle markers *Cdk1*, *Cdk2*, *Rpa1*, *Rabgap1*, and *App* were also expressed. From culturing the cells prior to RNA extraction and from PANTHER gene enrichment analysis and IPA pathway analysis, we have found that EPCs have the propensity to undergo cell proliferation and utilize canonical pathways that are important for germ cell development. This is an unexpected finding, since, after a proliferative phase, PGCs enter and arrest at the diplotene stage of prophase I of meiosis, thereby ending their proliferative capacity [38]. Moreover, we did not observe the expression of *Zp1*, *Zp2*, *Zp3* or *Figα*, which are required for differentiation to primordial oocytes [62, 63]. Therefore, we suggest that these EPCs are undifferentiated multipotent lineage-specific oogonial cells, that could differentiate into oocytes or be dedifferentiated under the right conditions. Interestingly, one of the top canonical pathways that was utilized by the EPCs was the mTOR signaling pathway, which is important for the maintenance of embryonic stem cells and is embryonically lethal when knocked-out [64]. We have also detected the expression of *c-kit*, which is a protein kinase receptor responsible for the reawakening of the quiescent primordial follicle to enter follicular growth, via the PI3K-AKT pathway [65], which is one of the top canonical pathway used, demonstrating the potential of EPCs to enter follicular development.

mtDNA is clonally amplified from ∼200 copies in PGCs to >150,000 copies in metaphase II oocytes [19, 41–43, 66, 67], which represents the potential number of molecules of the mitochondrial genome available for transmission to offspring. Two or more populations of mtDNA genotypes (wild type and molecules harboring variants) can co-exit, but variants normally exist at low levels in healthy individuals [21, 68]. Indeed, numerous studies have shown that pathogenic and non-pathogenic mtDNA variants are more prevalent in humans than previously thought [68–70]. Individuals remain healthy until pathogenic mtDNA variants pass a certain threshold, whereby wild-type mtDNA can no longer compensate for defective mtDNA [44]. Therefore, to ensure that EPCs possess the same mtDNA genotypes as oocytes, for the faithful transmission of germline mtDNA to offspring during assisted reproduction, we compared mtDNA variants harbored by mini-pig EPCs, oocytes, embryos, and ovarian tissues. We found that mtDNA sequence variants A376del, A1253del and A5188del were present in all samples at low percentages. On the other hand, the T7317C variant is harbored by the EPCs and oocytes at high percentages, but was not detected in embryos or ovarian tissues. To this end, our data on A376del, A1253del and A5188del indicate that oocytes and EPCs originate from the same lineage during early development and are recycled from one generation to the next as indicated by their presence in gametes, embryos and tissues. However, it appears that the T7317C variant was diluted out during embryo development [41, 43, 71].

From human studies and our mini-pig model, it has been shown that certain mtDNA variants tend to accumulate at a higher percentage in specific tissues [21, 69, 70]. In the present work, we found that the variants 1497InsA and T960C may have resulted from replication errors made by POLG [72], or were preferentially amplified during early embryo development [71], but they were not observed in the adult ovarian tissues. Only the variant A376del is consistently detected across EPCs, oocytes, embryos, as well as somatic tissues such as ovarian tissues, heart and brain [21], which suggests that this variant arose from the germline and is maintained in both germ cells and somatic tissues. Nevertheless, we found that the *de novo* acquisition of mtDNA variants is not very common in our mini-pig model. Therefore, our results indicate that EPCs harbor variants that originated from the germline. In addition, we suggest that it is important to faithfully transmit those variants to offspring, since they may be advantageous during development, and/or for maintaining natural genetic variation in the population [21]. Whilst mouse models possessing two genetically divergent non-pathogenic mtDNA genotypes have perturbed physiological functions [73], the exact role of endogenous non-pathogenic mtDNA variants is still unclear. Furthermore, our mini-pig model is not known to carry any mitochondrial disease causing mutations.

Accumulation of mtDNA variant load is associated with aging and other age-related disorders [44, 74]. There is also evidence to suggest that mtDNA variant load increases in oocytes and cumulus cells of women over the age of 35 [9, 75, 76]. To this end, EPCs represent an ideal population of cells for mitochondrial isolation to be used in the clinic, as they are genetically identical to the patient and harbor a low percentage of mtDNA variants. Since the proportion of mtDNA variants has been shown to increase in culture after each passage [77], we cultured the EPCs for one week without passage. This is beneficial for clinical applications, as a proportion of the viable EPCs could be used to screen for pathogenic mtDNA variants prior to mICSI. In this respect, EPCs are also a source of “ovarian stem cells”, or for generating “oocyte-like cells” to be used for ovarian transplantation. We found that mtDNA copy number of EPCs is within the previously reported range for PGCs [41–43]. This suggests that the mitochondria they reside in have low mitochondrial metabolic activity, as is the case for stem-like cells that primarily rely on glycolysis for energy production [78]. Furthermore, in agreement with our copy number data, EPCs express significantly fewer *Polg* transcripts compared with heart tissues.

In conclusion, we have characterized the gene expression profiles of EPCs by RNA-sequencing and performed gene enrichment analysis and pathway analysis to determine that EPCs possess proliferative and self-renewal capacity. The main aim of the current study was to determine whether EPCs are a suitable source to harvest naïve mitochondria to be used in mitochondrial supplementation during mICSI. We have achieved this aim by showing that EPCs possess mtDNA variants that are distinctive to the germline lineage. This unique population of cells could be used for *in vitro* maturation or ovarian transplantation to allow women with low ovarian reserve and/or hormone sensitivity to conceive. Furthermore, characterization of ovarian stem cells is important for our fundamental understanding of ovarian biology and the process of ovarian ageing.

## MATERIALS AND METHODS

### Animal ethics approval

Tissues obtained from mini-pigs were excess to requirement. Animals were euthanized in accordance with animal ethics guidelines. Approval for the use of animals was granted by Monash Medical Centre Animal Ethics Committee A, approval number MMCA/2012/84.

### Preparation of ovarian cortical strips from porcine tissue

Porcine ovaries were transported to the laboratory in sterile phosphate buffered saline (Sigma-Aldrich, St Louis, MO, U.S.A), and maintained at ∼38°C. Ovaries were cut in half lengthways and transferred to a sterile 10 cm dish containing phosphate buffered saline with penicillin and streptomycin. Avoiding the central cortex area, bisected ovaries were cut into thin slices using a carbon steel single edge razor blade. A size 10 or 11 scalpel blade was used to cut the ovary slices into strips, and then each strip was cut into small pieces. Approximately 30 pieces of ovarian tissue were washed and transferred to a cryovial containing 1ml of sterile, 90% FBS/10% DMSO freezing solution to be frozen overnight at −80°C, then transferred to LN_2_ tank for storage.

### Fluorescence-activated cell sorting

Porcine ovarian cortex tissue was digested and processed into a single cell suspension, based on methods described previously [48]. In brief, cells were resuspended and blocked in 2% human serum albumin (HSA) in HBSS (without Mg^2+^ and Ca^2+^) for 20 min at room temperature with agitation followed by an incubation with Alexa Fluor^®^ 647-conjugated anti-DDX4 antibody (HuMab DDX4) for 20 min at room temperature (in the dark) at a concentration of 10 μg per million cells per 100 μl. The cell suspension was washed by centrifugation in HBSS (without Mg^2+^ and Ca^2+^) followed by incubation with SYTOX^®^ green dead cell stain (Cat # S34860, ThermoFisher) at 30 μM for 20 min at room temperature with agitation (in the dark). For each experiment, an aliquot of unstained cells was used as the negative threshold and gating control. Labeled cells were filtered (35 μm pore diameter) and subjected to analysis on an SH800 flow cytometer with the manufacturer's SH800 software (Sony Biotechnology Inc., San Jose, CA, USA). Freshly isolated DDX4 positive viable cells (EPCs) were collected and frozen in cryopreservation buffer.

### Porcine EPC culture

The cells derived from porcine ovarian cortical strips were cultured in EPC media consisting of DMEM, supplemented with 10% FBS (heat inactivated), 1 mM sodium pyruvate, 1 mM nonessential amino acids, 1X penicillin-streptomycin-glutamine, 0.1 mM β-mercaptoethanol (all ThermoFisher Scientific), 10^3^ units/ml ESGRO leukemia inhibitory factor (LIF), 10 ng/ml recombinant human epidermal growth factor (rhEGF), 1 ng/ml basic fibroblast growth factor (bFGF), 40 ng/ml glial cell-derived neurotropic factor (GDNF; all Merck Millipore) and 1X N-2 MAX supplement (R & D Systems). On thawing, cells were plated at a density of 2.5 × 10^4^ /cm^2^ in EPC media at 39°C and 5% CO_2_ with media changes every 2 days. Once confluent, cells were lysed directly in the wells, lysates collected, snap frozen in LN_2_ and stored at −80°C until processed further for RNA extraction using the RNeasy Mini Kit (Qiagen), as per manufacturer's instructions.

### RNA library preparation and sequencing

Library preparation was performed using the TruSeq RNA v2 (Illumina, CA, USA) protocol. Briefly, mRNA was purified using oligo(dT) beads, and then fragmented. The 1^st^ strand cDNA synthesis was randomly primed followed by 2^nd^ strand cDNA synthesis. Sequencing adaptors were ligated and the library was amplified by PCR. RNA sequencing was performed on a HiSeq 3000 (Illumina). Sequencing data were deposited in the sequence read archive (SRA) in NCBI under the project number PRJNA374593.

### RNA sequencing bioinformatics analysis

46 ± 0.9 (mean ± S.E.M) million paired reads were obtained from RNA sequencing. Read quality was determined by the Illumina quality score, with >92% bases above Q30 across all five samples. Adaptor and overrepresented sequences were removed before the sequence reads were aligned to the pig reference genome (NCBI version: GCF_000003025.5_Sscrofa10.2). Using the Stringtie tool v 1.0.4, 79.7 ± 0.4 (mean ± S.E.M) % of the paired reads were mapped to the *Sus scrofa* exons. Data normalization was performed using edgeR. 13,808 normalized and annotated genes were identified. To determine the overall functions of EPCs, normalized RNA-Sequencing data were analyzed using the PANTHER classification system (http://www.pantherdb.org/) [79]. Ingenuity Pathway Analysis (IPA) (QIAGEN Redwood City, www.giagen.com/ingenguity, fall 2016 release), which is a web-based application, was used to determine pathways utilized by the EPCs. “Commonly Expressed Genes” tool was applied to the dataset (n = 5) to identify common genes. A further cutoff of 5-log ratio was applied and 4391 genes were used for pathway analysis to identify highly expressed genes.

### Real time PCR for estimation of mtDNA copy number

Each PCR reaction consisted of 2 μL of template DNA, 10μL of 2x SensiMix (Bioline), 1 μL of 5 μM of each forward (5′-CTCAACCCTAGCAGAAACCA-3′) and reverse primer (5′-TTAGTTGGTCGTATCGGAATCG-3′), and 6 μL of ultrapure ddH20, performed in a Rotergene-3000 real time PCR machine (Corbett Research, Cambridge, UK). A series of 10-fold dilutions (1 ng/μL to 1x 10-8 ng/μL) was used as the known standards. mtDNA quantification was determined from the standard curve, and mtDNA copy number was calculated based on the PCR product length.

### Reverse transcriptase PCR

RNA from oocytes, blastocysts and EPCs was isolated using the ARCTURUS® PicoPure® RNA Isolation Kit (ThermoFisher Scientific), as per manufacturer's instructions. First strand cDNA synthesis was performed using the qScript Flex cDNA kit (Quantabio, MA, U.S.A), according to manufacturer's protocol. The resultant cDNA was used to amplify target genes by PCR (Primer sequences; Supplementary Table 1). The expression of *Ddx4* was confirmed by Sanger sequencing using a previously described protocol [66].

### mtDNA amplification and purification

The whole mitochondrial genome from EPC isolates (n=4), immature oocytes (n=17), 2-cell embryos (n=2), 4-cell embryos (n=2), 8-cell embryos (n=2) and ovarian tissues (n=5) were amplified by long PCR, as previously described [66]. Briefly, 40 ng DNA, with 1× High Fidelity PCR buffer, 100 mM MgSO4, 1 mM dNTPs (Bioline), 1U of Platinum *Taq* High Fidelity (Invitrogen, CA, USA) and 10 μM of each forward and reverse primer (Primer sequences; Supplementary Table 1). PCR products were separated on a 0.7% agarose gel and purified using the QIAquick PCR Purification Kit (Qiagen, West Sussex, UK).

### Whole mitochondrial genome sequencing

The DNA concentration of purified long PCR products was determined by Qubit® dsDNA HS Assay kit (Invitrogen). For each sample, equal amounts of DNA were pooled from PCR product A and B (∼5 ng combined). DNA shearing was performed by sonication using the S220 Focused-ultrasonicator (Covaris, MA, USA) to generate a mean library size of ∼400 bp. Libraries were prepared with Ovation Ultralow system V2 (protocol M01380v1) (Nugen, CA, USA). 14 cycles of amplification were performed. Sequencing was performed using the 250 bp paired-end chemistry on the Illumina MiSeq v2 platform with PhiX spike-in for technical control. The MiSeq run generated a total of 22.8 million reads that passed filter. Each of the four samples generated 785,599 ± 22390 (mean ± S.E.M) reads.

### Identification of mtDNA sequence variants

Two FASTQ files for each sample were imported into CLC Genomics Workbench v9.5.1 for quality trimming. Duplicate reads were removed before the remaining reads were mapped to a reference pig mitochondrial genome AJ002189 [12] to generate a representative sequence. Read sequences were then mapped to the representative sequence without masking, with an insertion and deletion cost of 3 and minimum of 80% identity to the representative sequence. The low frequency variant detection tool was used to determine the level of sequence variants. Variant calling was made using the following parameters: 3% minimum threshold, presence of variant on forward and reverse reads. Each variant identified, had a minimum count of 140, within minimum sequence coverage of 4000.

### Statistical analysis

Data analysis was performed using GraphPad Prism v6.0f (GraphPad Software, Inc., CA, USA). mtDNA copy number between EPCs and immature oocytes was compared using Mann-Whitney test. *Polg* expression amongst EPCs, and heart and muscle tissues was compared using ordinary one-way ANOVA followed by Dunn's multiple comparisons test.

## SUPPLEMENTARY MATERIALS TABLE


