# Supplementary material for: The molecular characterization of porcine egg precursor cells
Source: Oncotarget. 2017 Jun 28;8(38):63484–505. doi: 10.18632/oncotarget.18833 (PMC5609938; doi:10.18632/oncotarget.18833)
Supplement: Supplementary file 1 [file oncotarget-08-63484-s001.pdf]

The molecular characterization of porcine egg precursor cells

SUPPLEMENTARY MATERIALS

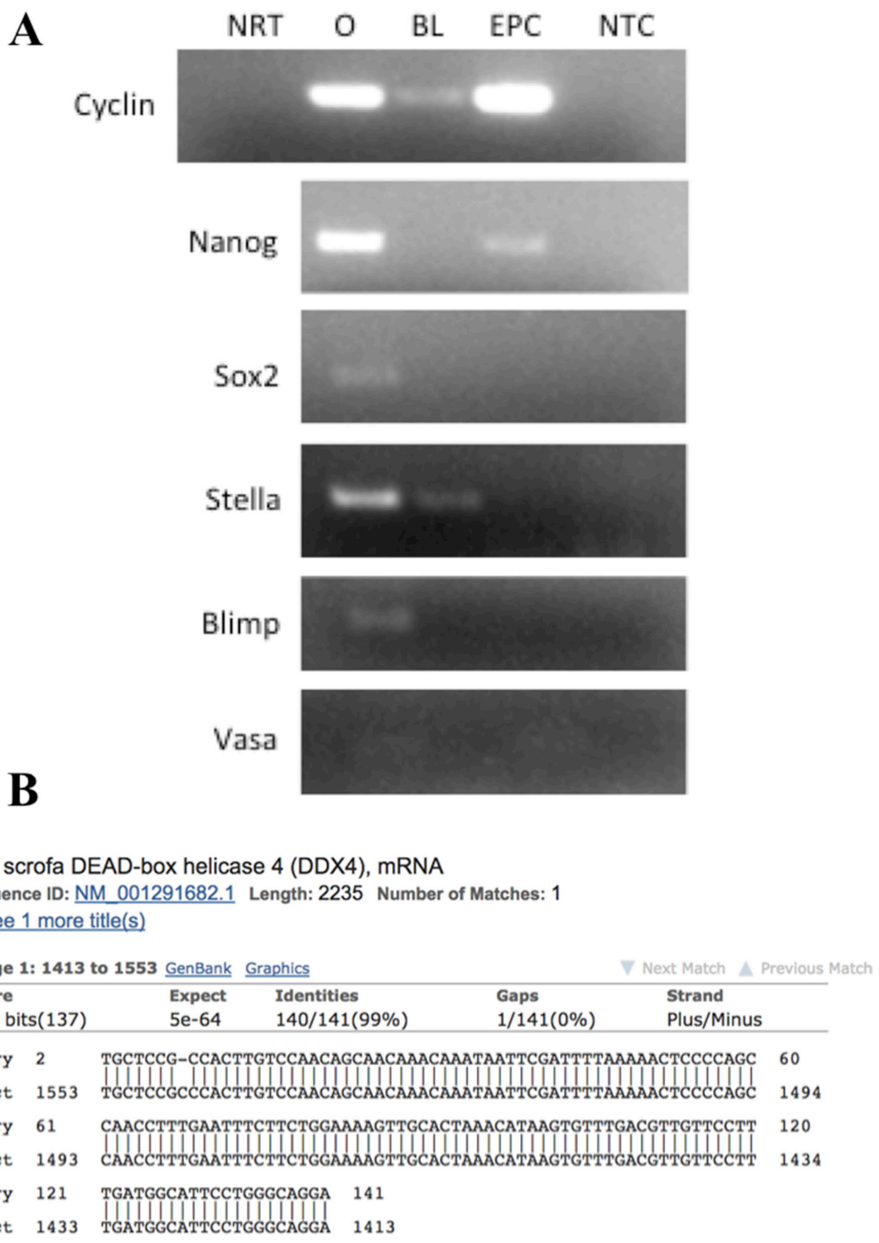

Supplementary Data 1: RT-PCR for *Cyclin*, *Nanog Sox2*, *Stella*. NRT = cDNA synthesis no reverse transcriptase control; O = oocyte; BL = blastocyst; EPC = egg precursor cells; NTC = PCR no template control **(A)**, and NCBI Blast result to confirm the expression of *Ddx4* **(B)**.

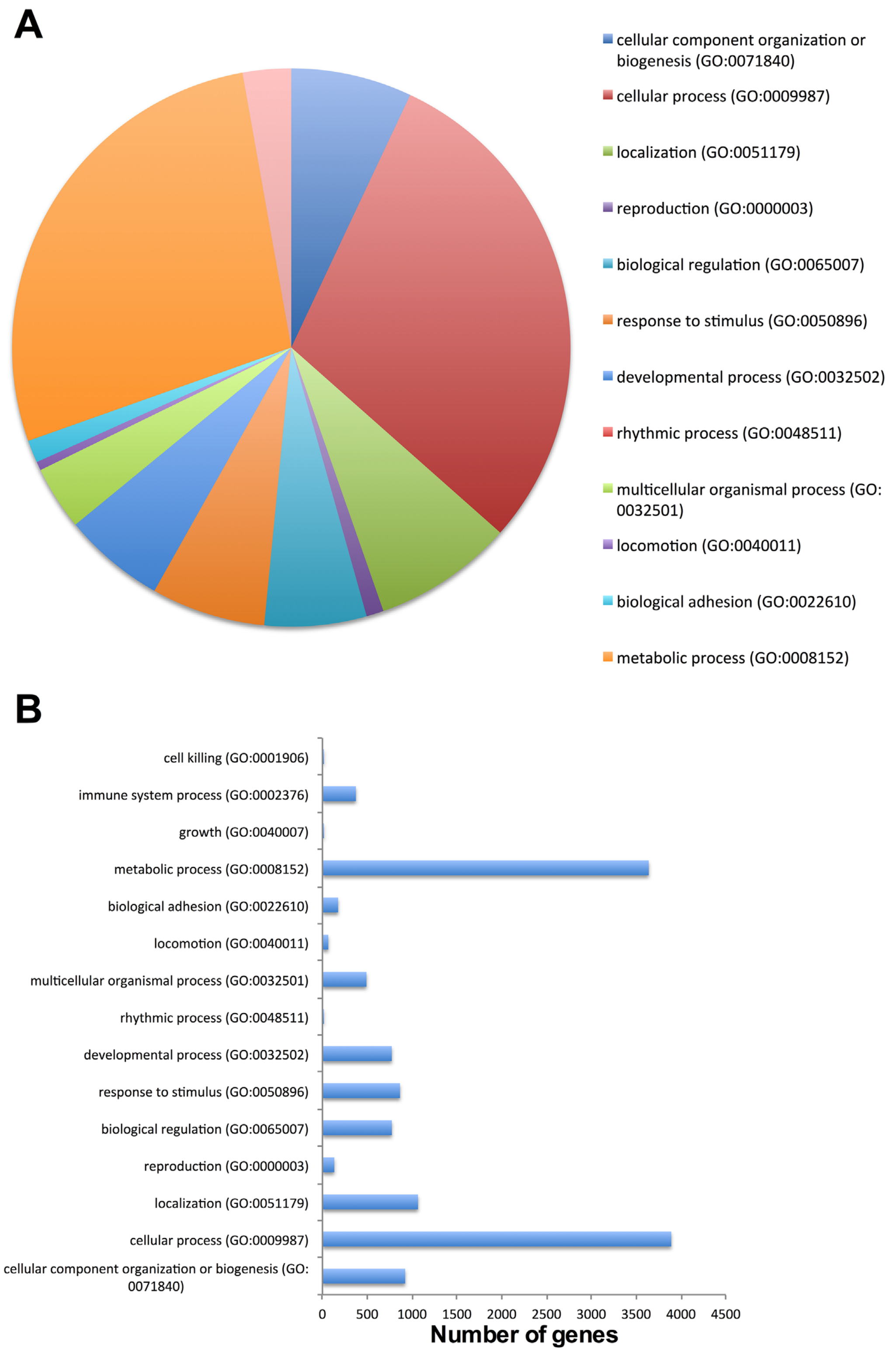

Supplementary Data 2: Top biological processes, as determined by the PANTHER classification system from the Gene Ontology Consortium database **(A)**, and the number of genes involved **(B).**

Supplementary Data 3: Top canonical pathways, as determined by IPA

See Supplementary File 1

Supplementary Data 4: IPA summary of top biological functions

See Supplementary File 2

Supplementary Data 5: Upstream regulators, as determined by IPA

See Supplementary File 3

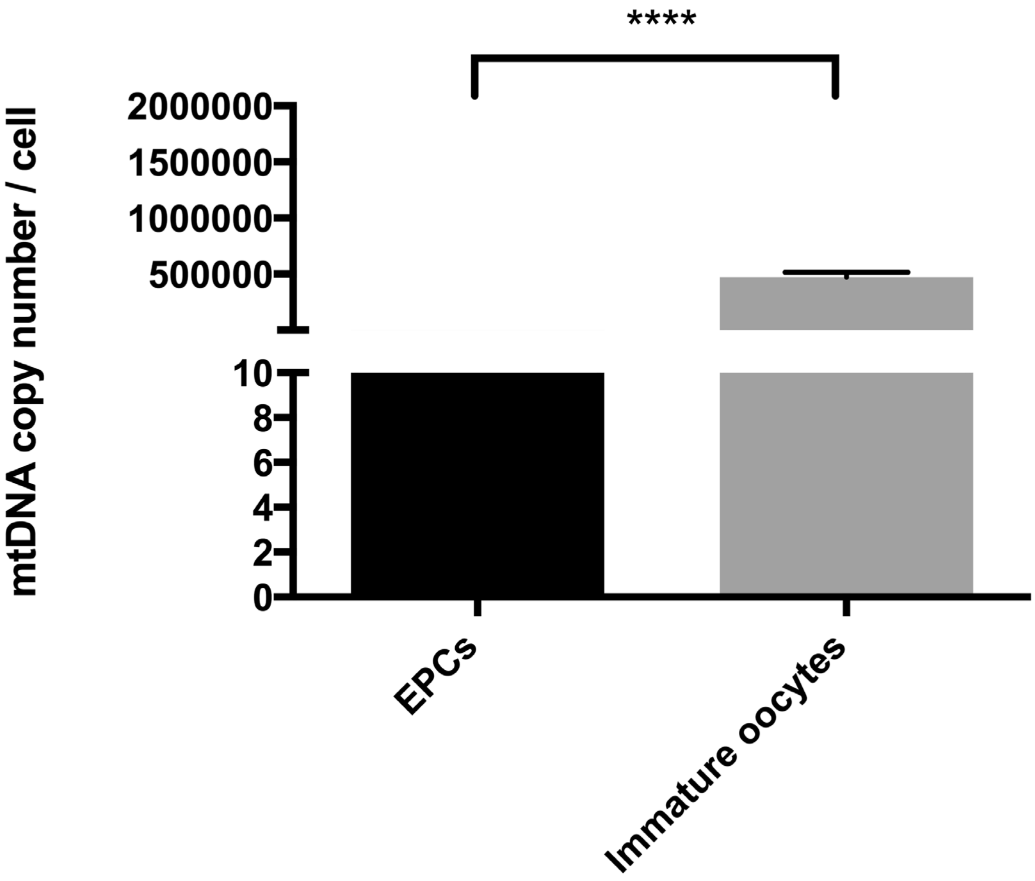

Supplementary Data 6: mtDNA copy number of EPCs compared with immature oocytes.

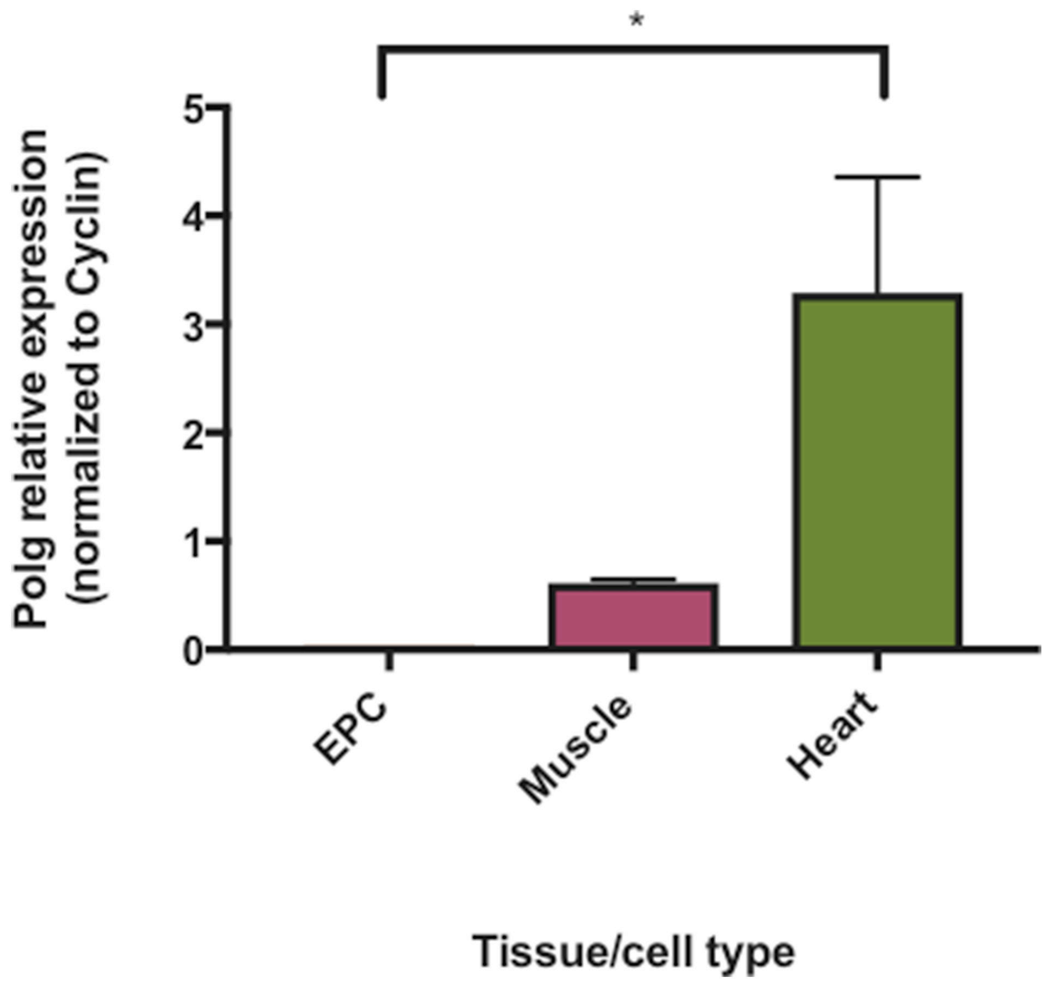

Supplementary Data 7: *Polg* gene expression. Values represent relative gene expression to *Cyclin* for each tissue or cell type.

Supplementary Table 1: Primer sequences, annealing temperatures and product sizes for PCR or qPCR reactions

Gene symbol	Accession number	Forward primer (5' to 3')	Reverse primer (5' to 3')	Annealing temp	Product length
mtDNA (Long A)	AJ002189	ATAGGACTCGAACCTAAACCTGAGAA	GACGAATAGTGCTACGGGAATGAATA	60	8272
mtDNA (Long B)	AJ002189	TTCTACCACTACTACTACTGACCTTA	AGAATATAGGAGGTTGATGATGATGG	60	9262
mtDNA	AJ002189	CTCAACCCTAGCAGAAACCA	TTAGTTGGTCGTATCGGAATCG	60	296
CYCLIN	NM_001170768.1	GTCGTGAAGTCACTGGAAAC	CCATCTGCCTGATTTGGTAC	60	276
SOX2	NM_001123197.1	AACCAGAAGAACAGCCCAGA	CGGGGCCGGTATTTATAATC	58	246
OCT4	NM_001113060	CACCTCAGGTCGGAGTGG	AGCTTGGCAAATTGTTCGA	55	226
Nanog	NM_001129971.1	AGGGCTCAGCCAGTACAGAA	TGATTTTCCCCAGCAGTTTC	60	316
DPPA3	XM_005655688.2	CCCGCCTTTCAATCTGTCTCC	TCGCCGAACCGTGTATCGAA	58	219
PRDM1	XM_013986966.1	CAGTGCCGTGAAGTTTCCA	AAGGATGCCTCTGCCTGAAC	56	186
DDX4	NM_001291682.1	CCTGCCCAGGAATGCCATCA	ACTGGCCAACTTGGAGAATGGT	55	180
POLG	XM_001927064.4	CTTACAACGAGGTGGATGTC	GGGAGCTGATACGTTTATGG	60	219
